# Necdin Promotes Ubiquitin-Dependent Degradation of PIAS1 SUMO E3 Ligase

**DOI:** 10.1371/journal.pone.0099503

**Published:** 2014-06-09

**Authors:** Ibrahim Gur, Kazushiro Fujiwara, Koichi Hasegawa, Kazuaki Yoshikawa

**Affiliations:** Laboratory of Regulation of Neuronal Development, Institute for Protein Research, Osaka University, Suita, Osaka, Japan; German Cancer Research Center, Germany

## Abstract

Necdin, a pleiotropic protein that promotes differentiation and survival of mammalian neurons, is a member of MAGE (melanoma antigen) family proteins that share a highly conserved MAGE homology domain. Several MAGE proteins interact with ubiquitin E3 ligases and modulate their activities. However, it remains unknown whether MAGE family proteins interact with SUMO (small ubiquitin-like modifier) E3 ligases such as PIAS (protein inhibitor of activated STAT) family, Nsmce2/Mms21 and Cbx4/Pc2. In the present study, we examined whether necdin interacts with these SUMO E3 ligases. Co-immunoprecipitation analysis revealed that necdin, MAGED1, MAGEF1 and MAGEL2 bound to PIAS1 but not to Nsmce2 or Cbx4. These SUMO E3 ligases bound to MAGEA1 but failed to interact with necdin-like 2/MAGEG1. Necdin bound to PIAS1 central domains that are highly conserved among PIAS family proteins and suppressed PIAS1-dependent sumoylation of the substrates STAT1 and PML (promyelocytic leukemia protein). Remarkably, necdin promoted degradation of PIAS1 via the ubiquitin-proteasome pathway. In transfected HEK293A cells, amino- and carboxyl-terminally truncated mutants of PIAS1 bound to necdin but failed to undergo necdin-dependent ubiquitination. Both PIAS1 and necdin were associated with the nuclear matrix, where the PIAS1 terminal deletion mutants failed to localize, implying that the nuclear matrix is indispensable for necdin-dependent ubiquitination of PIAS1. Our data suggest that necdin suppresses PIAS1 both by inhibiting SUMO E3 ligase activity and by promoting ubiquitin-dependent degradation.

## Introduction

Necdin was originally identified as a hypothetical protein encoded by a gene transcript expressed in neurally differentiated P19 embryonal carcinoma cells [1]. Necdin is expressed abundantly in postmitotic cells such as neurons and skeletal myocytes [2]–[4] and moderately in embryonic neural stem/progenitor cells [5], [6]. Necdin interacts with many regulatory proteins including E2F family proteins and p53 [7]–[11] and promote survival and differentiation of neurons and neural stem/progenitor cells [5], [6], [10]–[13]. Thus, it is likely that necdin serves as a hub of protein-protein interaction networks for neuronal development.

Necdin is a member of the MAGE protein family bearing a large homologous region known as the MAGE homology domain (MHD) [14], [15]. Placental mammals possess >30 MAGE genes per genome [14], [15], whereas only a single MAGE gene has been identified in invertebrates such as the fruit fly (*Drosophila melanogaster*) [16] and non-mammalian vertebrates such as the zebrafish (*Danio rerio*) [17] and chicken (*Gallus gallus*) [18]. In yeasts, the MAGE protein has been identified as Nse3, a subunit of the SMC (structural maintenance of chromosomes) 5/6 complex involved in the homologous recombination-based repair of DNA damage [19]–[21]. Thus, it is speculated that mammalian MAGE genes evolved from a single ancestral MAGE gene [22], [23]. Non-mammalian MAGE genes in the fruit fly [24], [25], zebrafish [17] and chicken [18] are expressed during neurogenesis, suggesting that MAGE family genes are involved in neuronal development.

Mammalian MAGE family proteins are divided into two classes based on the sequence similarities of the MHDs and gene expression patterns [15]: Type I MAGEs such as MAGEA, MAGEB, and MAGEC subfamilies are expressed in cancer and male germ cells but not in normal cells, and their genes are located on chromosome X [26]. In contrast, Type II MAGEs such as necdin, necdin-like 2, MAGED, MAGEE, MAGEF, MAGEH, and MAGEL are expressed in normal cells including neural cells. Among Type II MAGE proteins, necdin, MAGEL2 [27] and necdin-like 2 [28] are encoded by the genes that are closely located on human chromosome 15. Human necdin (*NDN*) and *MAGEL2* genes are located at chromosome 15q11-12, a region responsible for the pathogenesis of the classic genomic imprinting-associated neurodevelopmental disorder Prader-Willi syndrome [27], [29]–[32]. Both necdin and MAGEL2 are expressed only from the paternal alleles and implicated in the neurodevelopmental significance based on the phenotypes of gene knockout mice [12], [27], [33], [34]. In contrast to necdin and its homologous MAGE proteins, there is limited information about biochemical functions of most MAGE family proteins.

Posttranslational modifications of proteins with ubiquitin and SUMO (small ubiquitin-related modifier) modulate their stability, intracellular localization, and biological function [35]. Furthermore, a crosstalk between ubiquitination and sumoylation plays key roles in the regulation of various cellular functions [36], [37]. Several human MAGE proteins bind to RING (really interesting new gene)-type ubiquitin E3 ligases and promote ubiquitination of their substrates [38], [39]. Thus, MAGE family proteins may serve as adaptor proteins that regulate protein degradation and turnover via the ubiquitin-proteasome pathway. Both ubiquitination and sumoylation utilize E1, E2, and E3 enzymes for their covalent modifications of target proteins [40]. Although numerous ubiquitin E3 ligases are involved in the specific substrate recognition, sumoylation relies on only a small number of E3 ligases such as PIAS family [41], Cbx4 (Chromobox homolog 4)/Pc2 (Polycomb 2) [42], Nsmce2 (non-SMC element 2)/Mms21 [43], [44], and RanBP2 (Ran-binding protein 2) [45]. However, there is little information about physical and functional interactions between MAGE proteins and SUMO E3 ligases in mammalian cells. These findings prompted us to investigate whether necdin interacts with these SUMO E3 ligases.

We report here that necdin interacts with PIAS1, a typical RING-type SUMO E3 ligase involved in various biological events [46], [47]. We demonstrate that necdin suppresses PIAS1 via two distinct mechanisms whereby necdin suppresses sumoylation of PIAS1 substrate proteins and promotes PIAS1 degradation in the ubiquitin-proteasome pathway. We also show that the amino (N)- and carboxyl (C)-terminal regions of PIAS1 affect necdin-dependent ubiquitination. The present results provide novel insights into the regulatory mechanism of PIAS SUMO E3 ligase family by necdin and other MAGE family proteins.

## Results

### MAGE proteins differentially interact with SUMO E3 ligases

**Figure 1:**
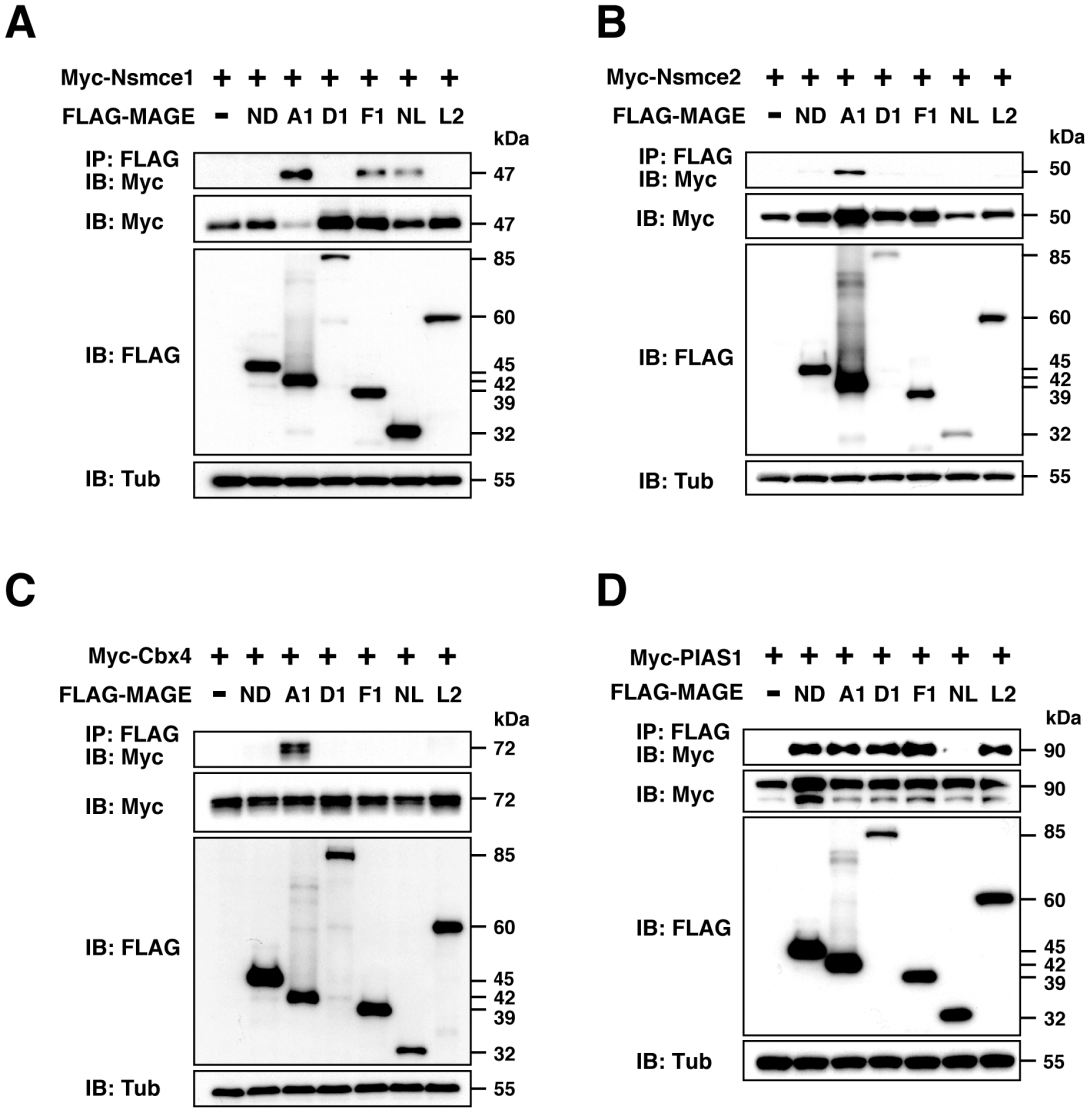
MAGE proteins differentially interact with Nsmce1, Nsmce2, Cbx4 and PIAS1. (**A**–**D**) Interactions of MAGE proteins with Nsmce1, Nsmce2, Cbx4, and PIAS1. Expression vectors for FLAG-tagged necdin (ND, 2 µg), MAGEA1 (A1, 1 µg), MAGED1 (D1, 8 µg), MAGEF1 (F1, 4 µg), necdin-like 2 (NL, 1 µg), and MAGEL2 (L2, 6 µg) were co-transfected with expression vectors for Myc-tagged Nsmce1 (Myc-Nsmce1, 3 µg)(**A**), Nsmce2 (Myc-Nsmce2, 3 µg)(**B**), Cbx4 (Myc-Cbx4, 3 µg)(**C**), and PIAS1 (Myc-PIAS1, 6 µg)(**D**) into HEK293A cells. Proteins were immunoprecipitated (IP) with anti-FLAG antibody (FLAG) and immunoblotted (IB) with anti-Myc (Myc) (top panels, **A**–**D**). Expressed proteins were immunoblotted (IB) with antibodies to Myc, FLAG and β-tubulin (Tub). Molecular sizes are in kilodaltons (kDa).

We analyzed the interactions of typical SUMO E3 ligases including Nsmce2, Cbx4 and PIAS1 with MAGE proteins including necdin, MAGEA1, MAGED1, MAGEF1, necdin-like 2 and MAGEL2 by co-immunoprecipitation assay. To verify the assay system, we used the RING-type protein (non-SMC element 1) which interacts with necdin-like 2 and MAGEF1 but not with necdin, MAGED1 and MAGEL2 [38]. We found that Nsmce1 interacted with MAGEA1, necdin-like 2 and MAGEF1, but not with necdin, MAGED1 or MAGEL2 (Fig. 1A). We then examined the interactions of the SUMO E3 ligases Nsmce2, Cbx4, and PIAS1 with these MAGE proteins (Fig. 1B–D). The MAGE proteins except MAGEA1 failed to interact with Nsmce2 and Cbx4 (Fig. 1B, C). Intriguingly, PIAS1 interacted with necdin, MAGEA1, MAGED1, MAGEF1 and MAGEL2, but not with necdin-like 2 (Fig. 1D). These data indicate that PIAS1 is a common target of diversified mammalian MAGE proteins.

### Necdin interacts with the central conserved region of PIAS1 via the MHD

**Figure 2:**
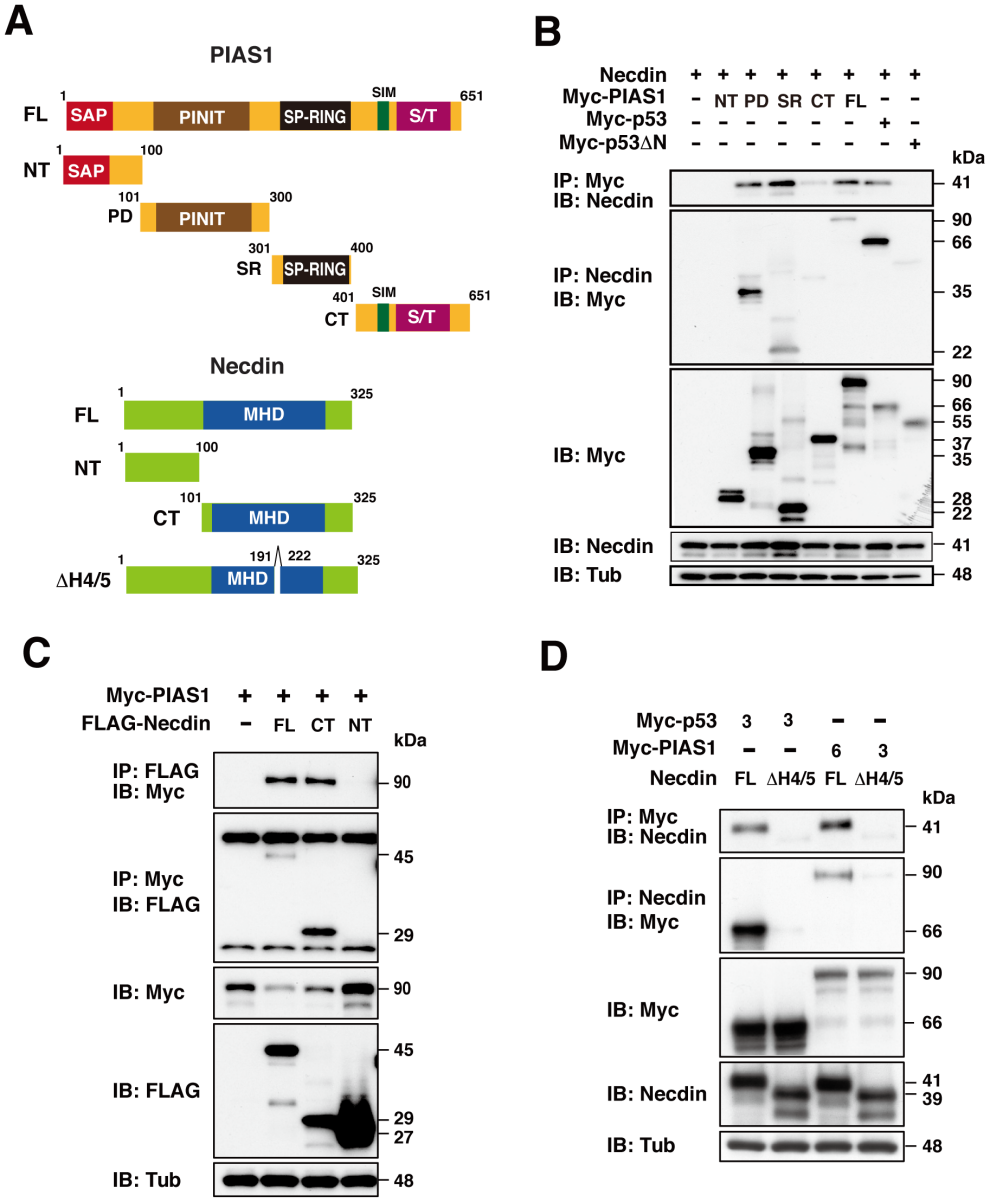
Necdin interacts with PIAS1 central conserved domains via the MHD. (**A**) Domain structures of mouse PIAS1 and necdin. Abbreviations: FL, full-length; NT, N-terminus; CT, C-terminus; SAP, SAF-Acinus-PIAS; PD, PINIT motif-containing domain; SR, SP-RING; SIM, SUMO-interacting motif; S/T, Ser/Thr-rich; MHD, MAGE homology domain; ΔH4/5, aa 191-222 deletion. (**B**) Interactions of necdin with PIAS1 deletion mutants. HEK293A cells were transfected with combinations of expression vectors for necdin (3 µg), and Myc-tagged PIAS1 mutants (NT, PD, SR, CT, FL; 3 µg each). Cell lysates were immunoprecipitated (IP) and immunoblotted (IB) with antibodies to Myc and necdin (top two panels). (**C**, **D**) Interactions of PIAS1 with necdin deletion mutants. HEK293A cells were transfected with expression vectors for Myc-tagged PIAS1 (Myc-PIAS1, 3 µg), FLAG-tagged necdin FL (1 µg), CT (1 µg), NT (8 µg) (**C**); Myc-p53 (3 µg), Myc-PIAS1 (3 or 6 µg), Necdin FL (1 µg), ΔH4/5 (1 µg) (**D**). Immunoprecipitated proteins were analyzed using antibodies to Myc (**C**, **D**), FLAG (**C**) and necdin (**D**)(top two panels). Expressed proteins were immunoblotted with antibodies to Myc, necdin, FLAG and γ-tubulin (Tub)(bottom three panels, **B**–**D**).

To characterize the interaction between necdin and PIAS1, we constructed deletion mutants of PIAS1 and necdin for co-immunoprecipitation assay using Human Embryonic Kidney 293A (HEK293A) cells (Fig. 2A). The PIAS1 mutants were designed to contain PIAS-specific motifs: PIAS1 amino acids (aa) 1-100 (NT) containing the SAF-Acinus-PIAS (SAP) domain; PIAS1 aa 101-300 (PD) containing the Pro-Ile-Asn-Ile-Thr (PINIT) domain; PIAS1 aa 301-400 (SR) containing the Siz/PIAS RING (SP-RING) domain; PIAS1 aa 401-651 (CT) containing the SIM (SUMO-interactive motif) and Ser/Thr-rich domains. We constructed necdin mutants such as necdin aa 1-100 (NT) containing N-terminal proline-rich acidic region, necdin aa 101-325 (CT) containing the entire MAGE homology domain (MHD) (aa 116-280) and necdin ΔH4/5 (aa 190-222 deletion) lacking the p53-interacting region [48], which corresponds to helices H4 and H5 region of necdin-like 2 MHD (PDB 3NW0) [38], [49]. Necdin was co-immunoprecipitated with the PIAS1 fragments containing the PINIT (PD) and SP-RING (SR) domains, and these were conversely co-immunoprecipitated with necdin (Fig. 2B). Necdin failed to interact with the PIAS1 terminal regions. In this assay, we confirmed that necdin bound to p53 (positive control) but not to N-terminally truncated p53 (p53 ΔN, negative control) [8].

We then analyzed the PIAS1-binding region of necdin using HEK293A cells transfected with truncated necdin mutants (Fig. 2C). PIAS1 interacted with full-length necdin and the N-terminally truncated mutant (CT) but not with the N-terminal region (NT). We also found that full-length necdin and CT reduced expression levels of Myc-PIAS1, suggesting that co-expressed necdin destabilizes the PIAS1 protein (Fig. 2C, third panel). To confirm whether the necdin MHD is required for PIAS1 binding, we used the p53 binding-defective mutant necdinΔH4/5 (Fig. 2D). This mutant failed to bind to PIAS1 or p53 (control), suggesting that the H4/5 region of the MHD is indispensable for the interaction between necdin and PIAS1.

### Necdin inhibits sumoylation of PIAS1 substrates

**Figure 3:**
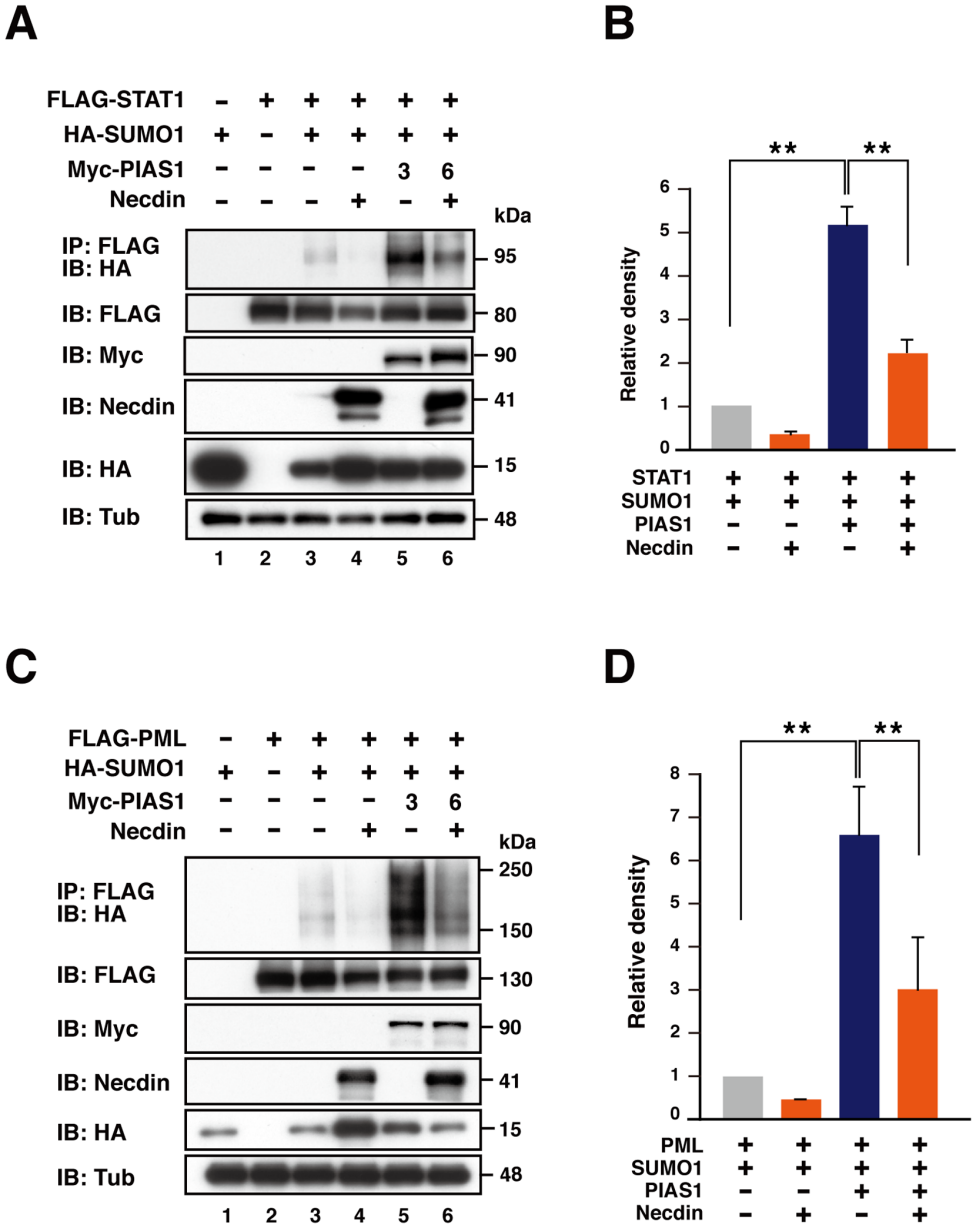
Necdin inhibits PIAS1-dependent sumoylation of STAT1 and PML. (**A**, **B**) Inhibition of PIAS1-dependent STAT1 sumoylation by necdin. HEK293A cells were transfected with combinations of expression vectors for FLAG-tagged STAT1 (FLAG-STAT1, 3 µg) (**A**), HA-tagged SUMO1 (HA-SUMO1, 10 µg), Myc-tagged PIAS1 (Myc-PIAS1, 3 or 6 µg) and necdin (Necdin, 1 µg). Cell lysates were immunoprecipitated (IP) with anti-FLAG antibody (FLAG) and immunoblotted (IB) with anti-HA antibody (HA). Expressed proteins were immunoblotted with antibodies to FLAG, Myc, necdin, HA, and γ-tubulin (Tub). Sumoylated STAT1 signals were quantified by densitometry and normalized with STAT1 (second panels) and PIAS1 levels (third panels)(**B**). (**C**, **D**) Inhibition of PIAS1-dependent PML sumoylation by necdin. HEK293A cells were transfected with combinations of expression vectors for FLAG-tagged PML (FLAG-PML, 3 µg), HA-tagged SUMO1 (HA-SUMO1, 4 µg), Myc-tagged PIAS1 (Myc-PIAS1, 3 or 6 µg) and necdin (Necdin, 1 µg). PML sumoylation was analyzed and quantified as above. Values (**B**, **D**) represent the mean ± SD (*n* = 3). **p<0.01.

Because necdin interacted with the PINIT and SP-RING domains that constitute the catalytic region of PIAS1 [50], we investigated whether necdin affects the SUMO E3 ligase activity of PIAS1. We examined the effects of necdin on PIAS1-dependent sumoylation using HEK293A cells transfected with SUMO1 and the PIAS1 substrates STAT1 [41] and PML [51]. Because the PIAS1 protein level was decreased by co-expressed necdin, we used twice the amount of the PIAS1-expressing vector to avoid reducing the PIAS1 enzyme level. In this assay, faint bands of sumoylated STAT1 were detected at ∼95 kDa in the absence of PIAS1, and PIAS1 promoted STAT1 sumoylation to 5.2 times the control level (Fig. 3A, B). Co-expression of necdin suppressed the PIAS1-promoted STAT1 sumoylation level by 57%. Similarly, PIAS1 markedly increased the level of sumoylated PML (6.6 times the control level) detected as smeared bands at >150 kDa, and co-transfection of necdin decreased the PML sumoylation level by 54% (Fig. 3C, D). These data suggest that necdin suppresses the SUMO E3 ligase activity of PIAS1.

### Necdin promotes PIAS1 degradation via the ubiquitin-proteasome pathway

**Figure 4:**
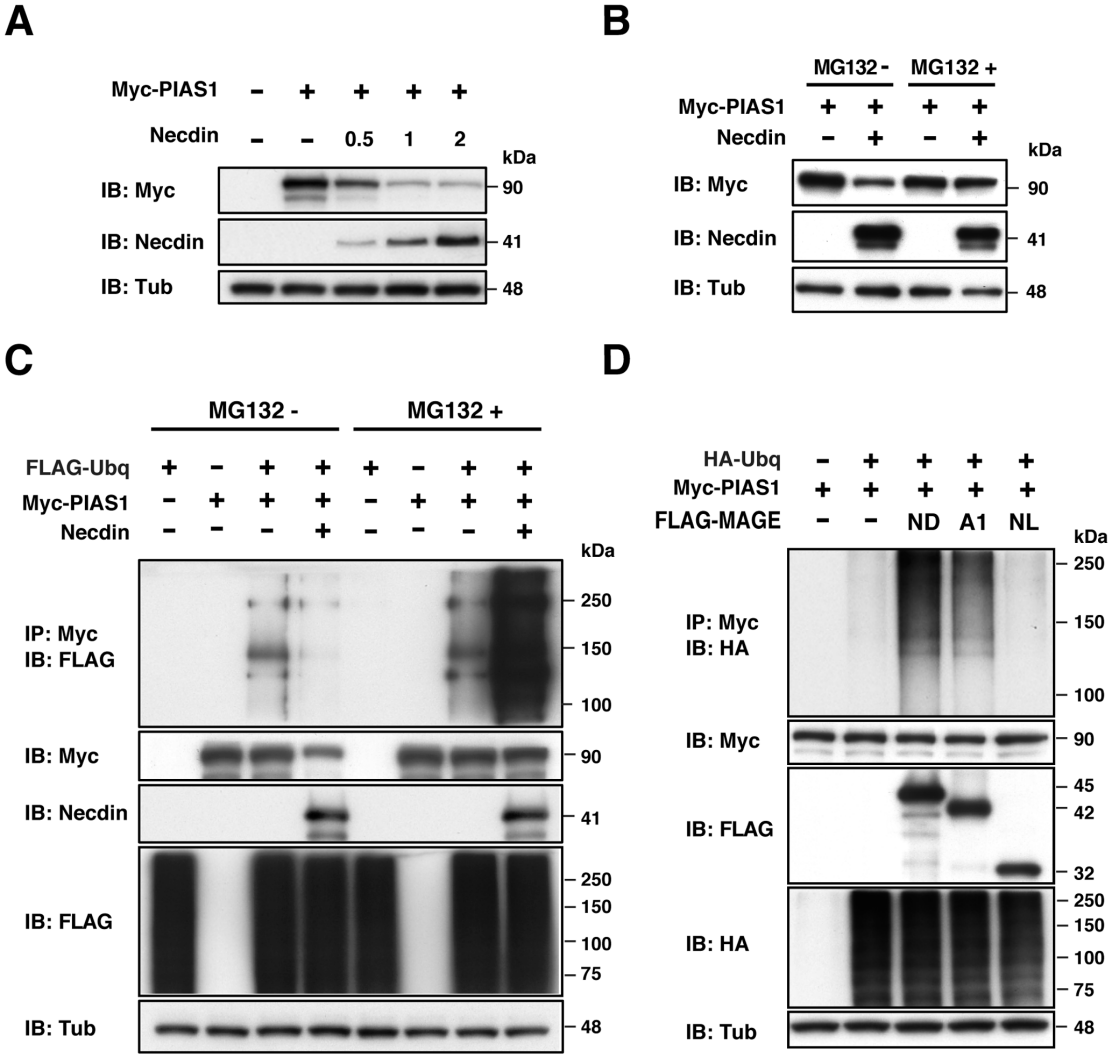
Necdin promotes PIAS1 degradation via the ubiquitin-proteasome pathway. (**A**) Necdin-dependent PIAS1 degradation. HEK293A cells were transfected with expression vectors for Myc-tagged PIAS1 (Myc-PIAS1, 3 µg) and necdin (0.5, 1, 2 µg). (**B**) Proteasome-mediated degradation of PIAS1. HEK293A cells were transfected with expression vectors for Myc-PIAS1 (3 µg) and necdin (1 µg), incubated for 20 hrs, treated with 30 µM MG132 (MG132+) or DMSO (MG132-) for 4 hrs, and harvested. Expression of Myc-PIAS1 (Myc), necdin and γ-tubulin (Tub) was analyzed by immunoblotting (**A**, **B**). (**C, D**) Necdin-promoted PIAS1 ubiquitination. HEK293A cells were transfected with combinations of expression vectors for FLAG-tagged ubiquitin (FLAG-Ubq, 4 µg)(**C**), HA-tagged ubiquitin (HA-Ubq, µg)(**D**), Myc-PIAS1 (3 µg), necdin (1 µg) (**C**) and FLAG-tagged necdin (ND, 2 µg), MAGEA1 (A1, 2 µg), and necdin-like 2 (NL, 1 µg) (**D**). Transfected cells were incubated for 20 hrs, treated with 30 µM MG132 for 4 hrs, and harvested. Cell lysates were immunoprecipitated (IP) with anti-Myc antibody (Myc) and immunoblotted (IB) with antibodies to FLAG (**C**) and HA (**D**). Expression of Myc-PIAS1 (Myc), necdin, FLAG-tagged proteins (FLAG), HA-Ubiquitin (HA) and γ-tubulin (Tub) was analyzed by immunoblotting. Abbreviations (**D**): ND, necdin; A1, MAGEA1; NL, necdin-like 2.

In the preceding experiments, PIAS1 protein levels were reduced when necdin was co-expressed. Thus, we examined whether necdin affects the stability of PIAS1 using transfected HEK293A cells (Fig. 4A). Necdin reduced the PIAS1 level in a dose-dependent manner. To examine whether necdin degrades the PIAS1 protein via the ubiquitin-proteasomal pathway, we used the proteasome inhibitor MG132. Treatment with MG132 protected PIAS1 from necdin-promoted degradation (Fig. 4B), suggesting that necdin promotes PIAS1 degradation in the proteasome.

We then examined whether necdin promotes PIAS1 ubiquitination by immunoprecipitation assay using HEK293A cells transfected with ubiquitin cDNA (Fig. 4C), Ubiquitinated PIAS1 was detected as multiple bands at 100–250 kDa. Although the ubiquitinated PIAS1 level was reduced, presumably owing to the degradation of the PIAS1 protein, in the absence of MG132, necdin significantly promoted PIAS1 ubiquitination in the presence of MG132 (Fig. S1A). We also found that the necdin ΔH4/5 mutant failed to promote PIAS1 ubiquitination (Fig. S1B). These data indicate that necdin promotes PIAS1 degradation in the ubiquitin-proteasome system. To analyze the specificity of necdin in PIAS1 degradation, we examined the effects of MAGEA1 and necdin-like 2 on PIAS1 ubiquitination in the presence of MG132. MAGEA1 promoted ubiquitination of PIAS1 to a significant but lesser extent than necdin, whereas necdin-like 2 exerted little or no ubiquitination-promoting effect (Fig. 4D, Fig. S1C).

**Figure 5:**
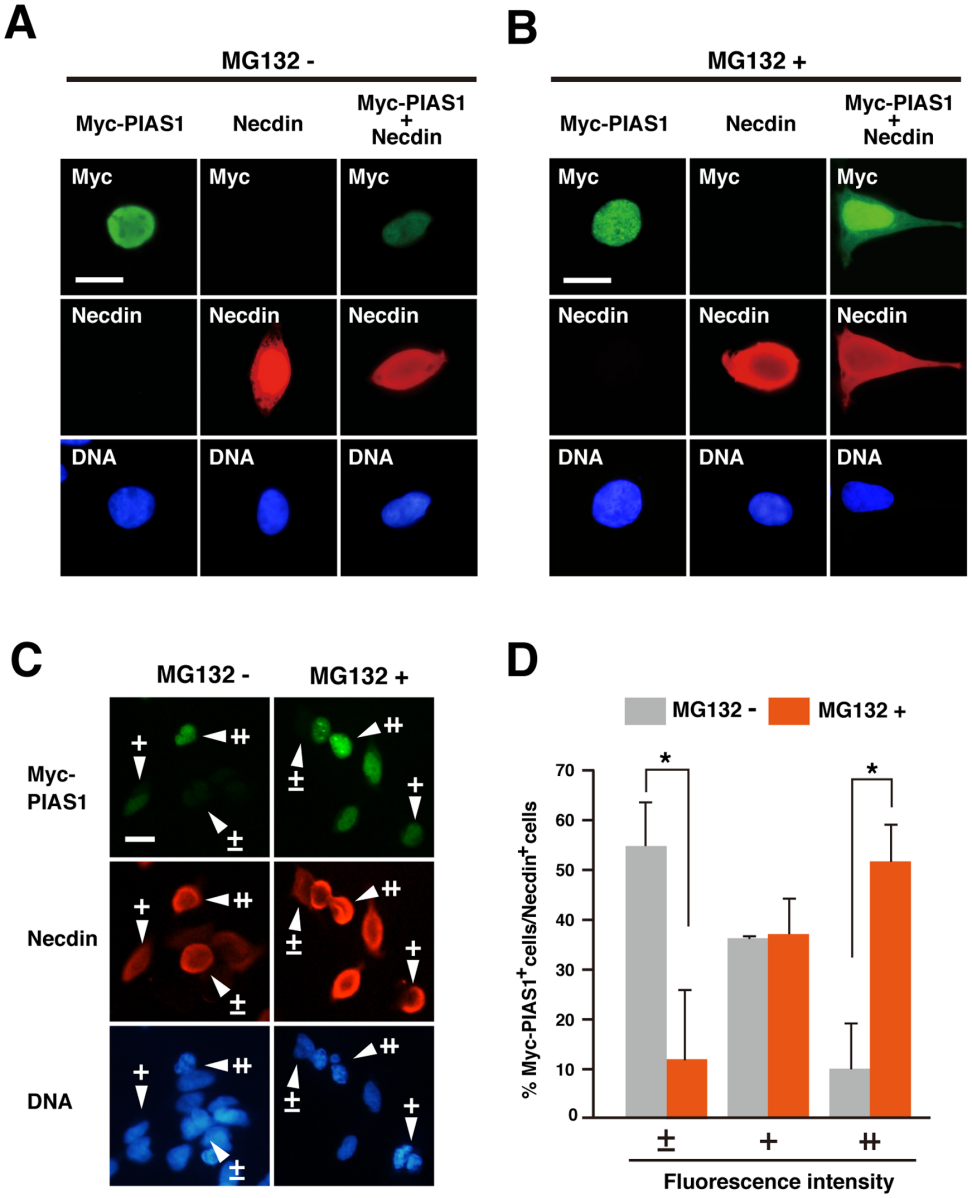
Necdin reduces nuclear PIAS1 levels in transfected cells. (**A**, **B**) Localization of PIAS1 and necdin in transfected cells. HEK293A cells were transfected with expression vectors for Myc-PIAS1 (Myc-PIAS1, 3 µg), necdin (Necdin, 2 µg) or both (Myc-PIAS1+Necdin) and fixed 24 hrs later (**A**). Transfected cells were treated with 30 µM MG132 for 4 hrs prior to harvest (**B**). Cells were immunostained with antibodies to Myc (top panels) and necdin (middle panels). Chromosomal DNA (DNA) was stained with Hoechst 33342 (bottom panels). (**C, D**) Quantification of PIAS1-expressing cells. Fluorescence intensities of PIAS1-expressing cells cotransfected with necdin were classified into three groups; undetectable or barely detectable (±), low (+), and high (++). Arrowheads point to representative cells (**C**). Classified PIAS1-expressing cells were quantified as % of necdin-expressing cells (**D**). Transfection efficiency (mean ± SD, *n* = 3): (MG132-) PIAS1, 24±4%; Necdin, 32±5%; PIAS1+Necdin, 32±5%; (MG132+) PIAS1, 27±1%; Necdin, 33±3%; PIAS1+Necdin, 39±4%. Values (**D**) represent the mean ± SD (*n* = 3). *p<0.05. Scale bars (**A**–**C**), 20 µm.

We next examined the subcellular localization of PIAS1 and necdin by immunocytochemistry using transfected HEK293A cells. PIAS1 was localized exclusively in the nucleus, whereas necdin was in both the nucleus and the cytoplasm (Fig. 5A). Although co-expression of necdin and PIAS1 did not alter their subcellular localization patterns, co-expressed necdin markedly reduced nuclear PIAS1 signals. We then examined the effects of MG132 on the distribution and expression levels of PIAS1 (Fig. 5B). MG132 *per se* had little or no effect on the localization of PIAS1 or necdin, but suppressed the necdin-induced reduction of nuclear PIAS1 levels. We classified PIAS1(Myc)-expressing cells into three groups by relative fluorescence intensities; undetectable or barely detectable, low, and high (Fig. 5C, D). The PIAS1 immunoreactivity in 55% of necdin-positive cells was undetectable or barely detectable in the absence of MG132, whereas 52% of necdin-immunopositive cells had high fluorescence intensities in the presence of MG132, suggesting that necdin promotes degradation of nuclear PIAS1 in the proteasome.

**Figure 6:**
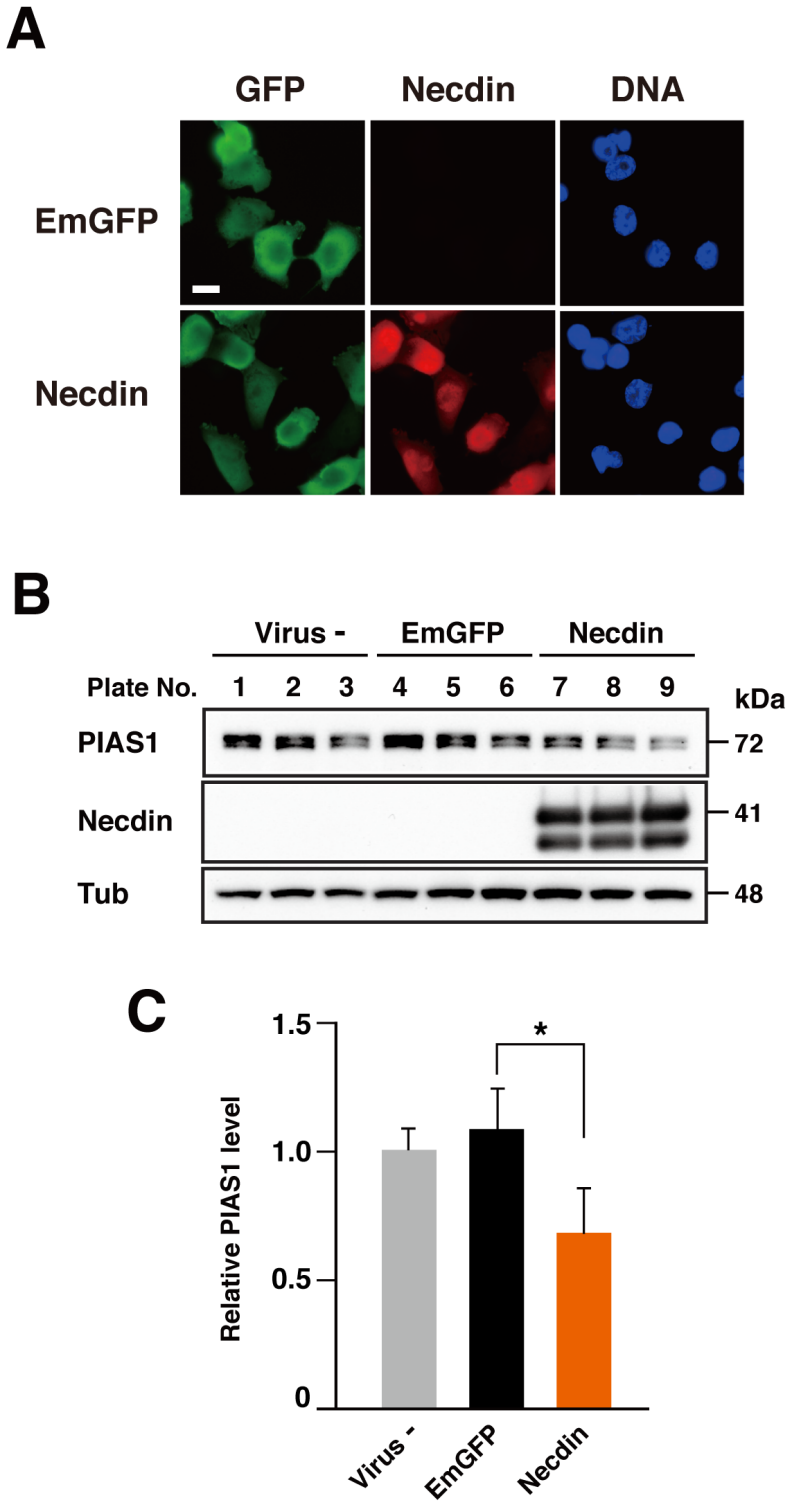
Lentivirus-mediated expression of necdin reduces endogenous PIAS1 levels. (**A**) H1299 cells infected with lentiviruses. Lentivirus vectors for EmGFP and necdin (+EmGFP) were infected into H1299 cells. Infected cells were grown on coverslips and fixed for immunocytochemistry using antibodies to GFP and necdin. Chromosomal DNA (DNA) was stained with Hoechst 33342. Scale bar, 20 µm. (**B**) Western blot analysis. Cell lysates of uninfected (Virus-, Plate Nos. 1–3) and infected cells expressing EmGFP (EmGFP, Plate Nos. 4–6) and necdin (Necdin, Plate Nos. 7–9) were analyzed 72 hrs after infection by immunoblotting using antibodies to PIAS1, necdin and γ-tubulin (Tub). (**C**) Quantification of the PIAS1 protein levels. Signal intensities of PIAS1 and γ-tubulin were quantitated by densitometry, and PIAS1 values were normalized with those of γ-tubulin. Values represent the mean ± SD (*n* = 3). *p<0.05.

To examine whether ectopic expression of necdin promotes degradation of endogenous PIAS1, we infected H1299 cells, a human non-small cell lung carcinoma cell line that endogenously expresses PIAS1 at a relatively high level, with necdin-expressing lentivirus vector and analyzed the PIAS1 level by Western blotting (Fig. 6). Under the conditions where most of the cells were infected with the control EmGFP vector (Fig. 6A), ectopic necdin significantly reduced the endogenous PIAS1 level in H1299 cells (Fig. 6B, C).

### PIAS1 N- and C-terminal regions are required for necdin-dependent ubiquitination

**Figure 7:**
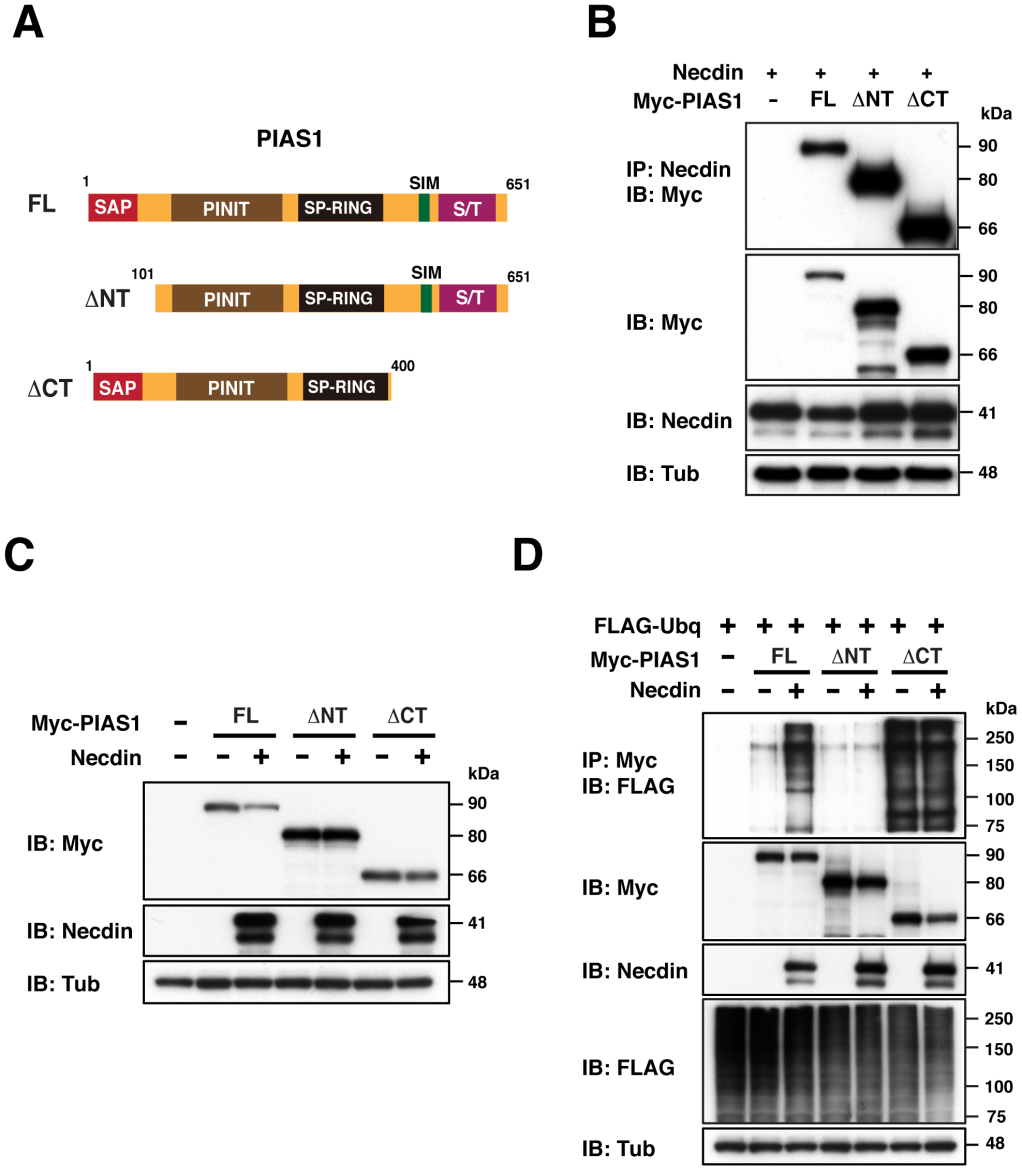
PIAS1 terminal-truncated mutants lack necdin-dependent ubiquitination. (**A**) Diagrams of PIAS1 terminal-truncated mutants. Abbreviations: FL, full-length; ΔNT, N-terminal deletion mutant; ΔCT, C-terminal deletion mutant. PIAS domain names are as in Fig. 2A. (**B**) Interactions of necdin with PIAS1 mutants. HEK293A cells were transfected with expression vectors for necdin (1 µg), Myc-tagged PIAS1 FL (6 µg), ΔNT (4 µg) and ΔCT (8 µg). Cell lysates were immunoprecipitated (IP) with anti-necdin antibody and immunoblotted (IB) with anti-Myc antibody. (**C**) Protein levels of PIAS1 terminal-truncated mutants. HEK293A cells were transfected with expression vectors for Myc-tagged PIAS1 mutants (FL, ΔNT, ΔCT; 3 µg each) and necdin (1 µg). (**D**) Ubiquitination of PIAS1 terminal-truncated mutants. HEK293A cells were transfected with expression vectors for FLAG-tagged ubiquitin (FLAG-Ubq, 4 µg), Myc-tagged PIAS1 mutants (3 µg), and necdin (1 µg), cultured for 20 hrs, and treated with 30 µM MG132 for 4 hrs prior to harvest. Cell lysates were immunoprecipitated (IP) with anti-Myc antibody (Myc) and immunoblotted (IB) with anti-FLAG antibody. Expression levels of Myc-PIAS1 mutants (Myc), necdin (Necdin), FLAG-ubiquitin (FLAG) and γ-tubulin (Tub) were analyzed by immunoblotting (**B**–**D**).

To determine the PIAS1 regions responsible for necdin-dependent degradation, we transfected HEK293A cells with PIAS1 mutants lacking the N-terminus (ΔNT) and C-terminus (ΔCT)(Fig. 7A). As expected, these truncated mutants, which contain the central conserved domains, bound to necdin (Fig. 7B). Although necdin significantly reduced the full-length PIAS1 level, PIAS1ΔNT and ΔCT mutants were resistant to necdin-promoted degradation (Fig. 7C, Fig. S1D). We also examined whether these mutants undergo necdin-dependent ubiquitination (Fig. 7D). PIAS1ΔNT was resistant to necdin-promoted ubiquitination, whereas PIAS1ΔCT underwent robust ubiquitination in a necdin-independent manner. These results suggest that these PIAS1 terminal regions mediate the susceptibility of PIAS1 to necdin-dependent ubiquitination.

**Figure 8:**
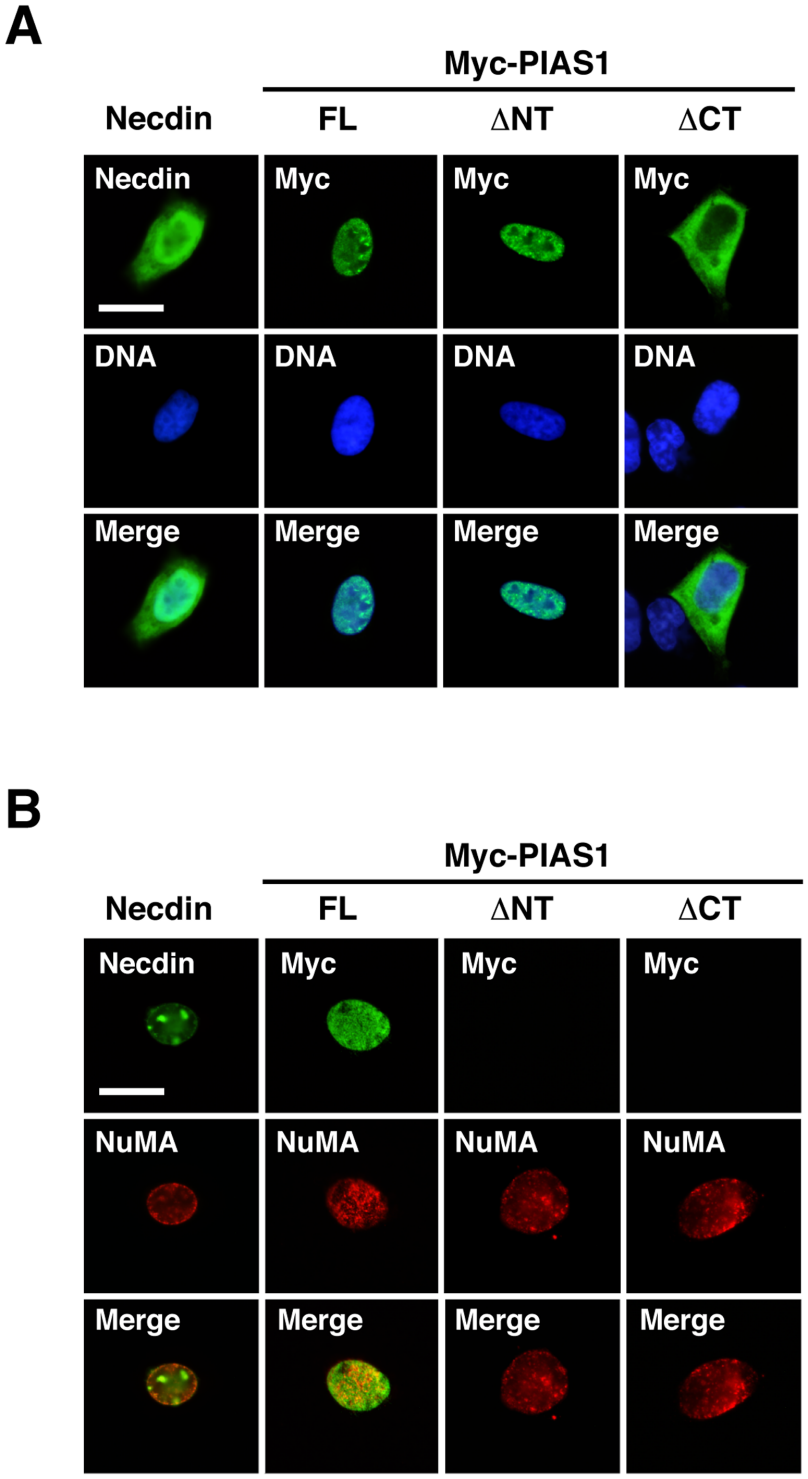
PIAS1 terminal-truncated mutants dissociate from the nuclear matrix. (**A**) Subcellular localization of necdin and PIAS1 terminal-truncated mutants. HEK293A cells were transfected with expression vectors for necdin (2 µg), Myc-tagged full-length PIAS1 and truncated mutants (FL, ΔNT, ΔCT; 3 µg each), fixed 24 hrs later, and immunostained with antibodies to necdin and Myc (top panels). Chromosomal DNA (DNA) was stained with Hoechst 33342 for nuclear location (middle panels). Images of expressed proteins and nuclear DNA are merged (bottom panels). Transfection efficiency (mean ± SD, *n* = 3): Necdin, 20±3%; FL,15±4%; ΔNT, 17±3%; ΔCT, 7±8%. (**B**) Localization of necdin and PIAS1 terminal-truncated mutants in the nuclear matrix. The nuclear matrix of HEK293 cells was prepared 24 hrs after transfection. Proteins associated with the nuclear matrix were double-stained for necdin (or Myc)(top panels) and the nuclear matrix protein NuMA (middle panels), and their images are merged (bottom panels). Scale bars (**A**, **B**), 20 µm.

To investigate the mechanism whereby the terminal truncation of PIAS1 affects necdin-dependent ubiquitination, we analyzed the subcellular localization of ectopically expressed PIAS1 mutants in HEK293A cells. The full-length PIAS1 and PIAS1ΔNT were distributed in the nucleus, whereas PIAS1ΔCT localized predominantly in the cytoplasm (Fig. 8A). All of the full-length and mutant PIAS1 (Myc)-transfected cells showed similar distribution patterns. PIAS1ΔNT partially accumulated as nuclear speckles that were immunopositive for PML (Fig. S2), consistent with the previous observation that a PIAS1 mutant lacking the SAP domain localizes to the PML bodies [52]. Because PIAS4 localizes in the nuclear matrix via its N-terminal SAP domain [53], we immunocytochemically examined the localization of these mutants in the nuclear matrix (Fig. 8B). Necdin localized in the nuclear matrix and accumulated as distinct speckles, consistent with the previous findings [54]. Full-length PIAS1 localized in the nuclear matrix of all of the transfected immunopositive cells, whereas PIAS1 deletion mutants were totally undetectable in the nuclear matrix, suggesting that the N- and C-terminal regions of PIAS1 are indispensable for its nuclear matrix localization.

## Discussion

The present study has demonstrated that necdin interacts with PIAS1 SUMO E3 ligase and suppresses its catalytic activity. Other MAGE proteins, except necdin-like 2, also bound to PIAS1, whereas Nsmce2 and Cbx4 SUMO E3 ligases bound only to MAGEA1 (Fig.1D). We have found that necdin interacts with RanBP2, another well-studied SUMO E3 ligase associated with the nuclear pore complex (KF and KY, unpublished observation). Several MAGE proteins bind to RING-type ubiquitin E3 ligases such as TRIM27 and TRIM28 to promote ubiquitination of their substrates, but necdin is unable to interact with these ubiquitin E3 ligases [38], [39]. We confirmed the previous findings [38], [55] that Nsmce1, the ubiquitin E3 ligase in the SMC5/6 complex, interacts with necdin-like 2 and MAGEF1 but not with necdin (Fig. 1A). Necdin-like 2 shows biochemical and functional characteristics similar to those of necdin [56]. Additionally, chicken and Drosophila MAGE proteins, which resemble mammalian necdin-like 2, exhibit functional similarities to necdin [18], [25]. In contrast to these similarities among MAGE family proteins, it is likely that mammalian MAGE proteins are diversified to interact with their specific ubiquitin and SUMO E3 ligases (Fig. S3).

The present study has also shown that necdin promotes degradation of PIAS1 via the ubiquitin-proteasome pathway. Necdin is likely to enhance PIAS1 ubiquitination by interacting with endogenous ubiquitin E3 ligases. PIAS1 is ubiquitinated by the ubiquitin E3 ligase hSiah2 (human homologues of seven in absentia) [57]. Necdin binds to the ubiquitin E3 ligase Mdm2 and promotes degradation of the proapoptotic protein CCAR1/CARP1 (cell cycle apoptosis regulatory protein) [58]. Thus, we examined whether hSiah2 and Mdm2 mediate necdin-promoted PIAS1 ubiquitination. However, we found that these ubiquitin E3 ligases were unable to ubiquitinate PIAS1 in a necdin-dependent manner (I.G. and K.Y., unpublished observations). Although detailed molecular mechanisms underlying necdin-promoted PIAS1 ubiquitination remain to be elucidated, we assume that necdin, like other MAGE proteins [38], serves as an adaptor to form a necdin-ubiquitin E3 ligase complex for PIAS1 ubiquitination. We found that PIAS1 promoted PML sumoylation (Fig. 3C), consistent with the previous report [51]. PIAS1-dependent PML sumoylation may promote PML degradation by RNF4, a SUMO-directed E3 ubiquitin ligase [51], [59], [60]. Thus, it is possible that necdin prevents PML degradation by suppressing PIAS1-dependent PML sumoylation. Because PML regulates cell fate during neocortical development [61], we speculate that necdin stabilizes PML by suppressing endogenous PIAS1 during neuronal differentiation.

The necdin-interacting region of PIAS1 consists of two known domains: the PINIT domain that contains the Pro-Ile-Asn-Ile-Thr motif in the middle of the PIAS-conserved sequence stretch [62] and the SP-RING domain similar to the RING-finger domain found in many ubiquitin E3 ligases [46], [63]. These two domains are highly conserved among PIAS family members [47]. Although PIAS1 interacts with SUMO-modified substrates via its distinct regions throughout the whole molecule [46], the SUMO ligase activity relies solely on these central domains [50]. As expected, necdin inhibited PIAS1-dependent sumoylation of STAT1 [41] and PML [51] (Fig. 3). Thus, it is likely that necdin-mediated suppression of the PIAS1 catalytic activity is attributed to its specific binding region in PIAS1.

The necdin deletion mutant that lacks aa 191-222 (ΔH4/5) in the MHD (aa116-280) failed to bind to PIAS1 (Fig. 2D), indicating that necdin interacts with PIAS1 via its MHD. Homology modeling based on the X-ray crystallographic data of necdin-like 2 [38] revealed that the mouse necdin aa 191-222 region corresponds to helices 4 and 5 in the winged-helix motif B (WH-B)(Fig. S4A, B). The H4/5 region in necdin-like 2 is predicted to form a hydrophobic pocket that interacts with NSMCE4A [49]. We have previously found that this deletion mutant neither induces cell growth suppression nor associates with the nuclear matrix [48]. Because MAGE proteins such as MAGEA1, MAGED1, MAGEF1 and MAGEL2 interact with PIAS1 (Fig. 1D), it is possible that the PIAS1-binding domains of these MAGE proteins take configurations similar to that of necdin. In contrast, necdin-like 2, which shows structural and functional similarities to necdin [56], failed to interact with PIAS1. Necdin-like 2 bears five amino acid substitutions in the H4/5 region compared with PIAS1-interacting MAGE proteins (Fig. S4C). It is noteworthy that three Thr residues are present in and near the H5 region of necdin-like 2, whereas other MAGE proteins contain only one or no Thr residue in their H4/5 regions. We speculate that necdin-like 2 lacks PIAS1 binding owing to its unique configuration of the H4/5 region.

The N-terminal deletion of PIAS1 lost both ubiquitination and responsiveness to necdin (Fig.7). The N-terminal domain contains a highly conserved SAP domain that mediates association with the nuclear matrix. Thus, PIAS1 is likely to interact with the nuclear matrix via the SAP domain. We found that the PIAS1 N-terminal deletion mutant was dissociated from the nuclear matrix and translocated to the PML bodies (Fig. 8B, Fig. S2), consistent with the previous report [52]. We have previously shown that necdin localizes in the nuclear matrix and interacts with the nuclear matrix-associated protein SAF-A/hnRNP U [54]. These findings suggest that necdin and PIAS1 are colocalized in the nuclear matrix, where necdin modulates PIAS1 ubiquitination. Intriguingly, PIAS1 C-terminal deletion enhanced ubiquitination in a necdin-independent manner (Fig. 7), implying that the PIAS1 C-terminus negatively controls necdin-promoted ubiquitination. Because C-terminally truncated PIAS1 was translocated to the cytoplasm (Fig. 8), the C-terminus of PIAS1 is likely to contribute to the nuclear retention. We speculate that these terminal regions are required for the translocation of PIAS1 to the nuclear matrix that contributes to the necdin-dependent ubiquitination.

Necdin plays anti-apoptotic and pro-survival roles in various cell types such as neurons and neural stem/progenitor cells through interactions with various proteins [5], [6], [8]–[12], [64]. Previous studies have shown that PIAS1 promotes apoptosis in response to various stimuli [52], [65], [66]. These observations suggest that necdin targets PIAS1 to suppress its proapoptotic activities. Because the necdin-binding region in PIAS1 is highly conserved among PIAS family members, it is possible that necdin also interacts with other PIAS family proteins to regulate their functions. Although information on the regulation of PIAS family proteins during neuronal development is currently limited, we speculate that necdin interacts with PIAS family proteins to modulate sumoylation of their target proteins involved in mammalian neuron development.

## Materials and Methods

### cDNAs and plasmids

Full-length cDNAs encoding mouse Nsmce1 (NCBI NM_026330.3), Nsmce2 (NCBI NM_026746.3), Cbx4/Pc2 (NCBI NM_007625.2) and PIAS1 (NCBI NM_019663.3) were cloned from mouse E14.5 forebrain cDNA library and subcloned into 6xMyc(N)-pcDNA3.1(+) vector [67]. Expression vectors for mouse p53, necdin, and their deletion mutants were constructed as described [5], [48]. cDNAs for mouse MAGED1, necdin-like2 and MAGEL2 were cloned as described [4], [56]. Human MAGEF1 was cloned from nuclear DNA library of HEK293A cells. Human MAGEA1 cDNA was provided by Dr. Kyogo Itoh (Kurume University School of Medicine). cDNAs encoding PIAS1 aa 1-100, 101–300, 301–400 and 401–652 or deletion mutants aa 101–652 and 1–400 were generated using synthetic oligonucleotide primers and subcloned into the expression vector. cDNAs encoding full-length mouse STAT1 (NCBI NM_001205313.1) and PML (NCBI NM_008884.5) were cloned from cDNA libraries of E14.5 mouse forebrain and adult mouse spleen, respectively, and subcloned into p3xFLAG-CMV10 (Sigma-Aldrich). cDNA encoding mouse SUMO1 was obtained from RIKEN BioResource Center DNA Bank and subcloned into hemagglutinin (HA)-pcDNA3.1(+) carrying the N-terminal HA sequence. All cDNAs used were sequenced for their identities. Experiments were approved by Recombinant DNA Committee of Osaka University (Approval No. 2938-1) and performed in accordance with national and institutional guidelines.

### Co-immunoprecipitation assay

HEK293A cells (1×10^6^ cells per 60 mm dish) were transfected with combinations of expression vectors, incubated in Dulbecco's Modified Eagle Medium (Life Technologies) supplemented with 10% fetal bovine serum at 5% CO_2_ at 37°C, and harvested 24 hrs after transfection as described [7]. The total amount of infected DNA was equalized by adding pcDNA3.1(+) vector. Transfected cells were lysed in a lysis buffer containing 10 mM Tris-HCl (pH 8.0), 150 mM NaCl, 1 mM EDTA, 1% IGEPAL CA-630 (Sigma-Aldrich), 10% glycerol, and protease inhibitors (Complete, Roche). Lysates (200 µg) were incubated at 4°C for 2 hrs with antibodies to FLAG (M2; Sigma-Aldrich; 1∶100), Myc (9E10; 1∶10) and necdin (NC243; 1∶200)[68]. Proteins bound to antibodies were pelleted with protein A-Sepharose (GE Healthcare), eluted from protein A-Sepharose, separated by 10% SDS-PAGE, and electroblotted onto polyvinylidene difluoride membranes (Immobilon; Millipore). Membranes were incubated with antibodies to Myc (1∶10), FLAG (1∶500), necdin (1∶3000), HA (HA.11; Covance; 1∶100), β-tubulin (TUB 2.1; Sigma-Aldrich;1∶1000) and γ-tubulin (GTU-88; Sigma-Aldrich;1∶1000) at room temperature for 1 hr. After incubation with horseradish peroxidase-conjugated anti-mouse and anti-rabbit IgGs (Cappel) for 1 hr at room temperature, proteins were visualized with chemiluminescence reagents (Western Lightning Plus-ECL, PerkinElmer).

### Sumoylation assay

HEK293A cells (1×10^6^ cells per 60 mm dish) were transfected with combinations of expression vectors for HA-SUMO1, Myc-PIAS1, necdin, FLAG-STAT1 and FLAG-PML and harvested 24 hrs after transfection. For analysis of PML sumoylation, cells were treated with 10 µM MG132 (Peptide Institute) 4 hrs prior to harvest as described [51]. Cells were lysed in the lysis buffer supplemented with 20 mM N-ethylmaleimide, and the lysates (300 µg) were incubated at 4°C for 2 hrs with anti-FLAG antibody (M2; Sigma-Aldrich). The protein-antibody complexes were pelleted with protein A-Sepharose, separated by 10% SDS-PAGE, and detected by Western blotting as above. Signal intensities of sumoylated proteins were quantitated by densitometry with ImageJ 1.48 software. The protein concentration was determined by the Bradford method (Bio-Rad).

### Ubiquitination assay

HEK293A cells (1×10^6^ cells per 60 mm dish) were transfected with combinations of expression vectors for FLAG-tagged ubiquitin, HA-tagged ubiquitin, Myc-tagged full-length PIAS1, Myc-tagged PIAS1 deletion mutants, necdin, FLAG-tagged necdin, FLAG-tagged MAGEA1, and FLAG-tagged necdin-like 2. Transfected cells were incubated for 20 hrs, treated with 30 µM MG132 or DMSO (vehicle control) for 4 hrs, and harvested. Cells lysates (250 µg) were incubated at 4°C for 2 hrs with the anti-Myc antibody, and the protein-antibody complexes were pelleted with protein A-Sepharose, separated by 10% SDS-PAGE, and detected by Western blotting as above. Signal intensities of ubiquitinated proteins were quantitated by densitometry.

### Immunocytochemistry

HEK293A cells (2–4×10^5^ cells per 35 mm dish) were cultured on coverslips for 17 hrs, transfected with expression vectors, and incubated for another 24 hrs at 5% CO_2_ at 37°C. Cells were fixed with 10% formalin for 20 min at room temperature and permeabilized with methanol for 20 min at room temperature. For proteasome inhibition, transfected cells were incubated in the presence of 30 µM MG132 or DMSO for 4 hrs prior to fixation. For immunostaining of the nuclear matrix-associated proteins, cells were sequentially treated with 0.5% Triton X100, 25 U/ml RNase-free DNase (RQ1, Promega), 0.25 M ammonium sulfate and 2M NaCl according to the method described [54], [69]. Fixed cells were treated with PBS-Tween 20 containing 1% BSA (Sigma-Aldrich) for 1 hr at room temperature, incubated with primary antibodies to Myc (Cell Signaling; 1∶300), necdin (NC243;1∶1000) and NuMA (Ab-1, Oncogene;1∶100) overnight at 4°C, and with Alexa Fluor 488-conjugated anti-mouse (or anti-rabbit) IgG (Life Technologies;1∶1000) and cyanine 3-conjugated anti-rabbit (or anti-mouse) IgG (Jackson ImmunoResearch, 1∶500). Chromosomal DNA was stained with 3.3 µM Hoechst 33342 (Sigma-Aldrich). Stained cells were observed with a fluorescence microscope (BX-50-34-FLAD1, Olympus), and images were captured with a charge-coupled device camera system (DP-70; Olympus).

### Lentivirus infection

Recombinant lentiviruses were produced in HEK293FT cells by transfecting SIN vector plasmids and two helper plasmids as described previously [70], [71]. Necdin cDNAs were subcloned into pENTR1A entry vector (Life Technologies) to construct CSII-EF1α-necdin-IRES-EmGFP, in which Emerald Green Fluorescent Protein (EmGFP) (Life Technologies) was used for an expression indicator [6]. The viral titer was measured by serial dilution on HEK293FT cells (Life Technologies) and determined as GFP-positive cell population by immunocytochemistry. Lentivirus vectors for EmGFP (empty vector) or necdin (+EmGFP) were infected into H1299 cells at a multiplicity of infection (moi) of 12, and the infected cells were incubated for 72 hrs prior to analyses. Expressed proteins were analyzed by Western blotting using antibodies against PIAS1 (EPR2518Y, Abcam; 1∶5000), necdin, and γ-tubulin. Signal intensities of proteins were quantitated by densitometry.

### Statistics

Statistical significance was tested using Tukey-Kramer multiple comparison method unless otherwise stated. A significance of *p*<0.05 was required for rejection of the null hypothesis.

## Supporting Information

Figure S1 **Quantification of necdin-promoted ubiquitination and degradation of PIAS1.** (**A**) Effects of necdin on PIAS1 ubiquitination. Ubiquitinated PIAS1 signals shown in Fig. 4C were quantified by densitometry, and the signal intensities of ubiquitinated proteins were normalized with those of Myc-PIAS1. Images with two different exposure times for each sample were analyzed. (**B**) Effect of necdin ΔH4/5 mutant on PIAS1 ubiquitination. PIAS1 was ubiquitinated by wild-type necdin (WT) and necdin ΔH4/5 mutant (ΔH4/5) in the presence of MG132, and ubiquitinated PIAS1 signals were quantified as above. (**C**) Effects of MAGEA1 and necdin-like 2 on PIAS1 ubiquitination. Ubiquitinated PIAS1 signals shown in Fig. 4D was ubiquitinated as above. (**D**) Effects of necdin on protein levels of PIAS1 and its mutants. Signals of ubiquitinated PIAS1 and its mutants shown in Fig. 7C were analyzed. Values (**A**–**D**) are mean ± SD (*n* = 3). *p<0.05, **p<0.01, ***p<0.001(by unpaired Student's *t* test in **D**). Abbreviations are as in Figs. 4 and 7.
(TIF)

Figure S2 **The PIAS1 N-terminal-truncated mutant localizes at the PML bodies.** HEK293A cells were transfected with expression vectors for Myc-tagged full-length PIAS1 (Myc-PIAS1, 3 µg) and N-terminal-truncated PIAS1 mutant (PIAS1ΔNT, 3 µg), fixed 24 hrs later, and double-stained for Myc-PIAS1 (Myc) and PML. Images of Myc and PML are merged (Merge). Arrows point to Myc-PIAS1^+^/PML^+^ speckles. Chromosomal DNA (DNA) was stained with Hoechst 33342. Scale bar, 10 µm.
(TIF)

Figure S3 **Summary of the interactions between MAGE proteins and SUMO and ubiquitin E3 ligases.** The interactions between MAGE proteins and SUMO E3 ligases (shown in Fig. 1B–D) are schematically presented (upper panel). Information on the interactions between MAGE proteins and ubiquitin E3 ligases was taken from the report by Doyle JM et al. [38] (lower panel). Abbreviations: ND, necdin; A1, MAGEA1; D1, MAGED1; F1, MAGEF1; NL, necdin-like 2; L2, MAGEL2; A2/3/6, MAGEA2/MAGEA3/MAGEA6.
(TIF)

Figure S4 **Protein structure modeling and primary sequence alignment of MAGE helices 4/5 regions.** (**A, B**) Structure models of the necdin H4/5 region. A structural model of the necdin MHD was created by Spanner homology modeling program using the crystal structure data of necdin-like 2 (PDB 3NW0). A view focused on the H4/5 region (necdin aa 191-222) in the winged-helix B area is presented. Helices 4 (H4) and 5 (H5), positions of 191(M) and 222 (G) (**A**), the hydrophobic pocket consisting of surface hydrophobic (red) and hydrophilic (blue) residues (**B**) are indicated. (**C**) Sequence alignment of the H4/5 region. Consensus amino acid residues (≥3 identical)(green), the residues unique to necdin-like 2 (red), and Thr (T)(dot) are shown. Abbreviations: ND, necdin; A1, MAGEA1; D1, MAGED1; F1, MAGEF1; L2, MAGEL2; NL, necdin-like 2.
(TIF)
