# Supplementary figures and images for: Necdin Promotes Ubiquitin-Dependent Degradation of PIAS1 SUMO E3 Ligase
Source: PLoS One. 2014 Jun 9;9(6):e99503. doi: 10.1371/journal.pone.0099503 (PMC4049815; doi:10.1371/journal.pone.0099503)

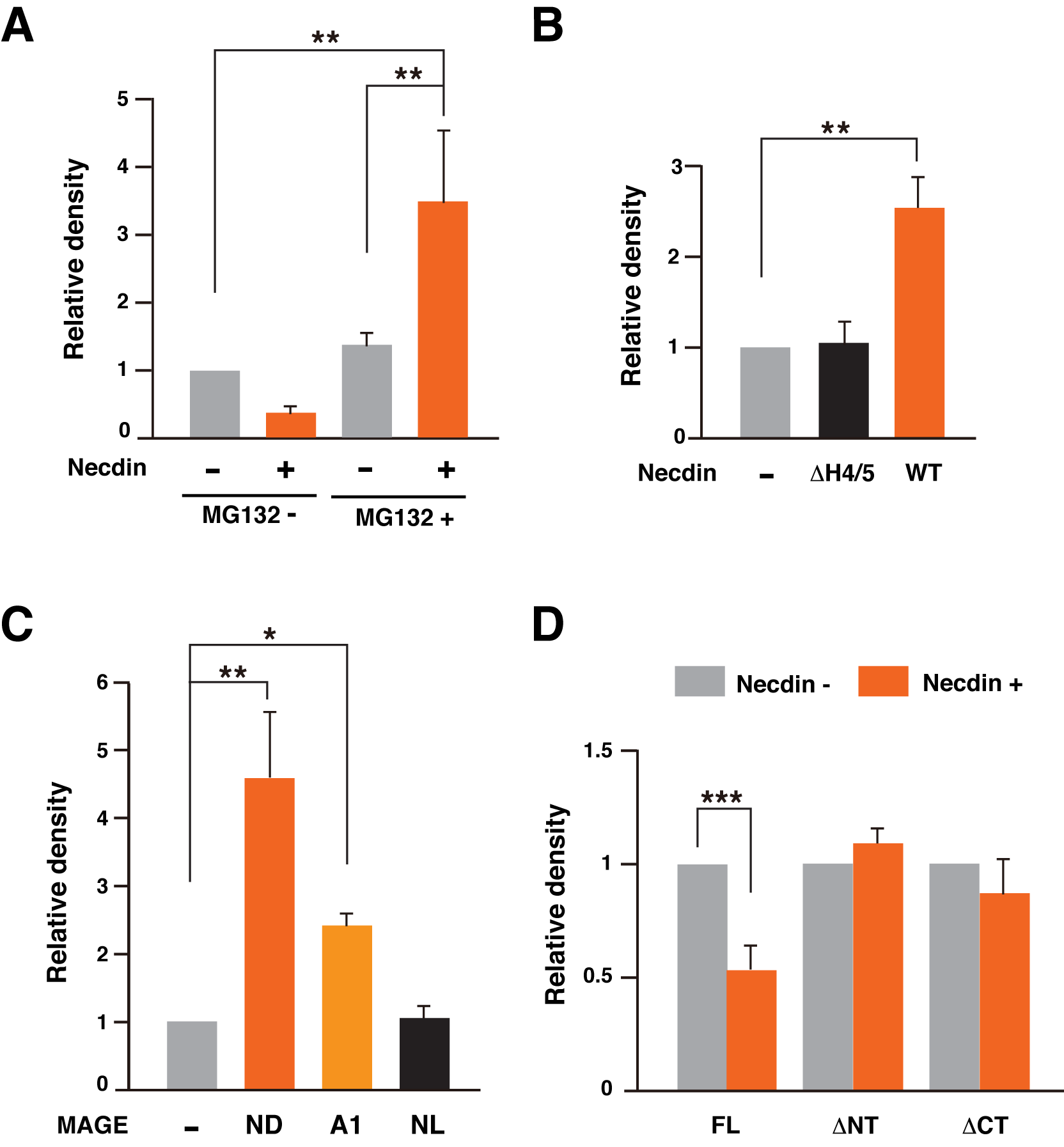

Supplement: Figure S1 — Quantification of necdin-promoted ubiquitination and degradation of PIAS1. (A) Effects of necdin on PIAS1 ubiquitination. Ubiquitinated PIAS1 signals shown in Fig. 4C were quantified by densitometry, and the signal intensities of ubiquitinated proteins were normalized with those of Myc-PIAS1. Images with two different exposure times for each sample were analyzed. (B) Effect of necdin ΔH4/5 mutant on PIAS1 ubiquitination. PIAS1 was ubiquitinated by wild-type necdin (WT) and necdin ΔH4/5 mutant (ΔH4/5) in the presence of MG132, and ubiquitinated PIAS1 signals were quantified as above. (C) Effects of MAGEA1 and necdin-like 2 on PIAS1 ubiquitination. Ubiquitinated PIAS1 signals shown in Fig. 4D was ubiquitinated as above. (D) Effects of necdin on protein levels of PIAS1 and its mutants. Signals of ubiquitinated PIAS1 and its mutants shown in Fig. 7C were analyzed. Values (A–D) are mean ± SD (n = 3). *p<0.05, **p<0.01, ***p<0.001(by unpaired Student's t test in D). Abbreviations are as in Figs. 4 and 7. (TIF) [file pone.0099503.s001.tif]

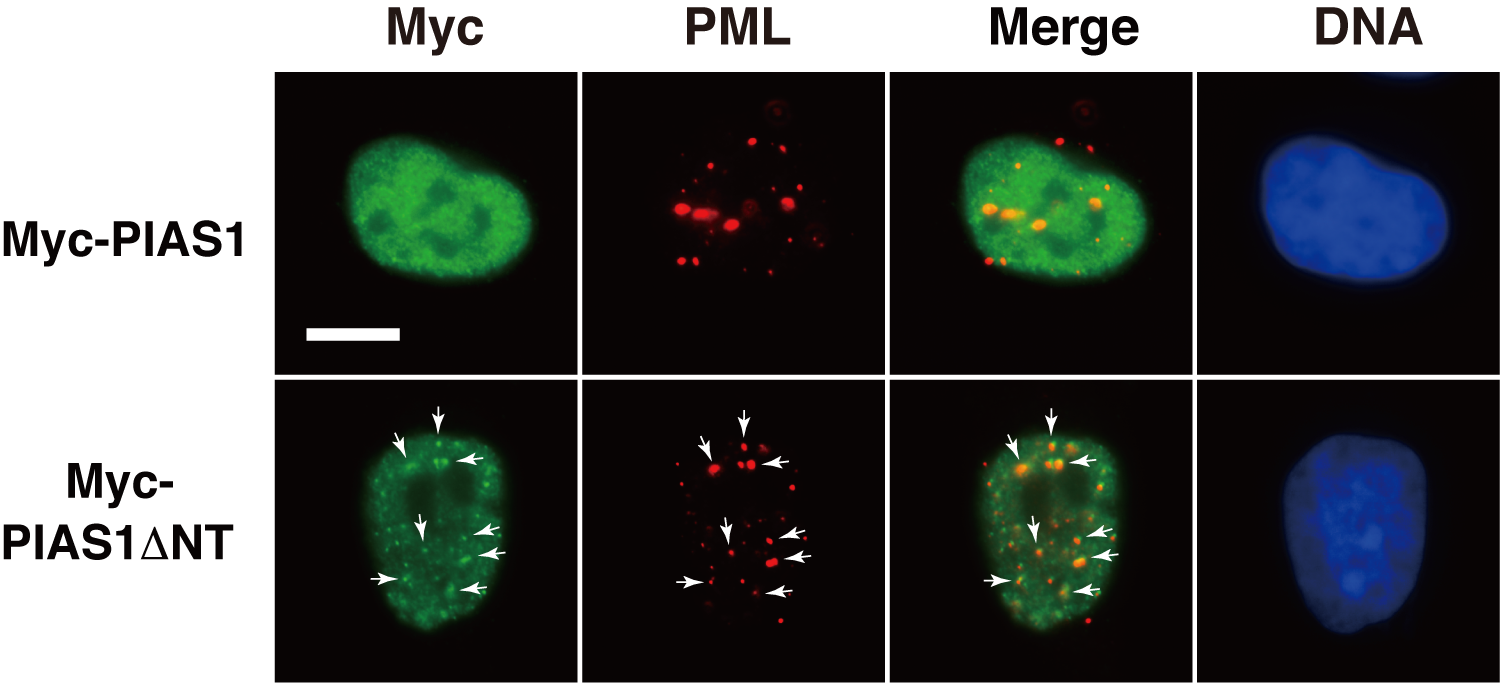

Supplement: Figure S2 — The PIAS1 N-terminal-truncated mutant localizes at the PML bodies. HEK293A cells were transfected with expression vectors for Myc-tagged full-length PIAS1 (Myc-PIAS1, 3 µg) and N-terminal-truncated PIAS1 mutant (PIAS1ΔNT, 3 µg), fixed 24 hrs later, and double-stained for Myc-PIAS1 (Myc) and PML. Images of Myc and PML are merged (Merge). Arrows point to Myc-PIAS1+/PML+ speckles. Chromosomal DNA (DNA) was stained with Hoechst 33342. Scale bar, 10 µm. (TIF) [file pone.0099503.s002.tif]

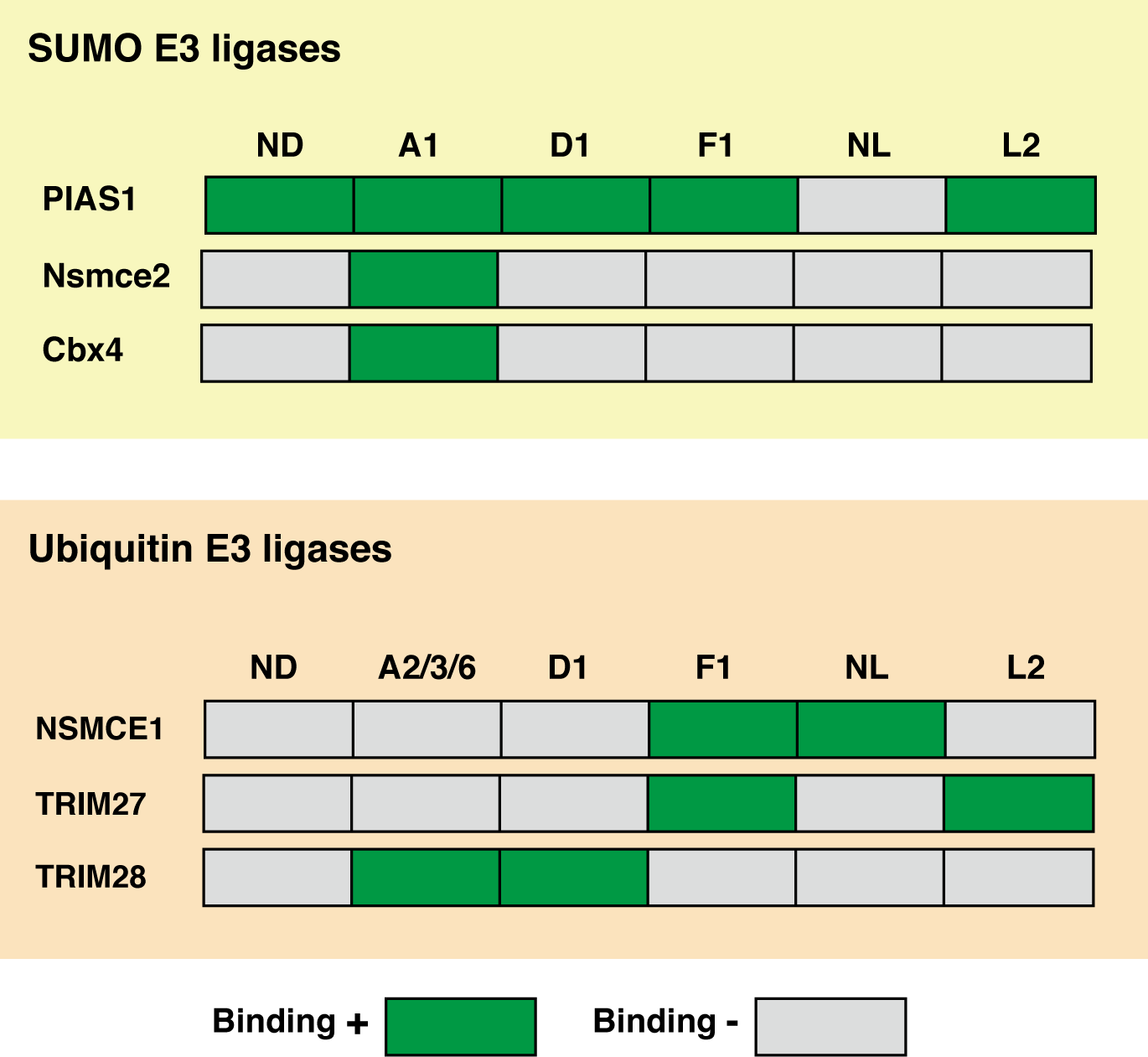

Supplement: Figure S3 — Summary of the interactions between MAGE proteins and SUMO and ubiquitin E3 ligases. The interactions between MAGE proteins and SUMO E3 ligases (shown in Fig. 1B–D) are schematically presented (upper panel). Information on the interactions between MAGE proteins and ubiquitin E3 ligases was taken from the report by Doyle JM et al. [38] (lower panel). Abbreviations: ND, necdin; A1, MAGEA1; D1, MAGED1; F1, MAGEF1; NL, necdin-like 2; L2, MAGEL2; A2/3/6, MAGEA2/MAGEA3/MAGEA6. (TIF) [file pone.0099503.s003.tif]

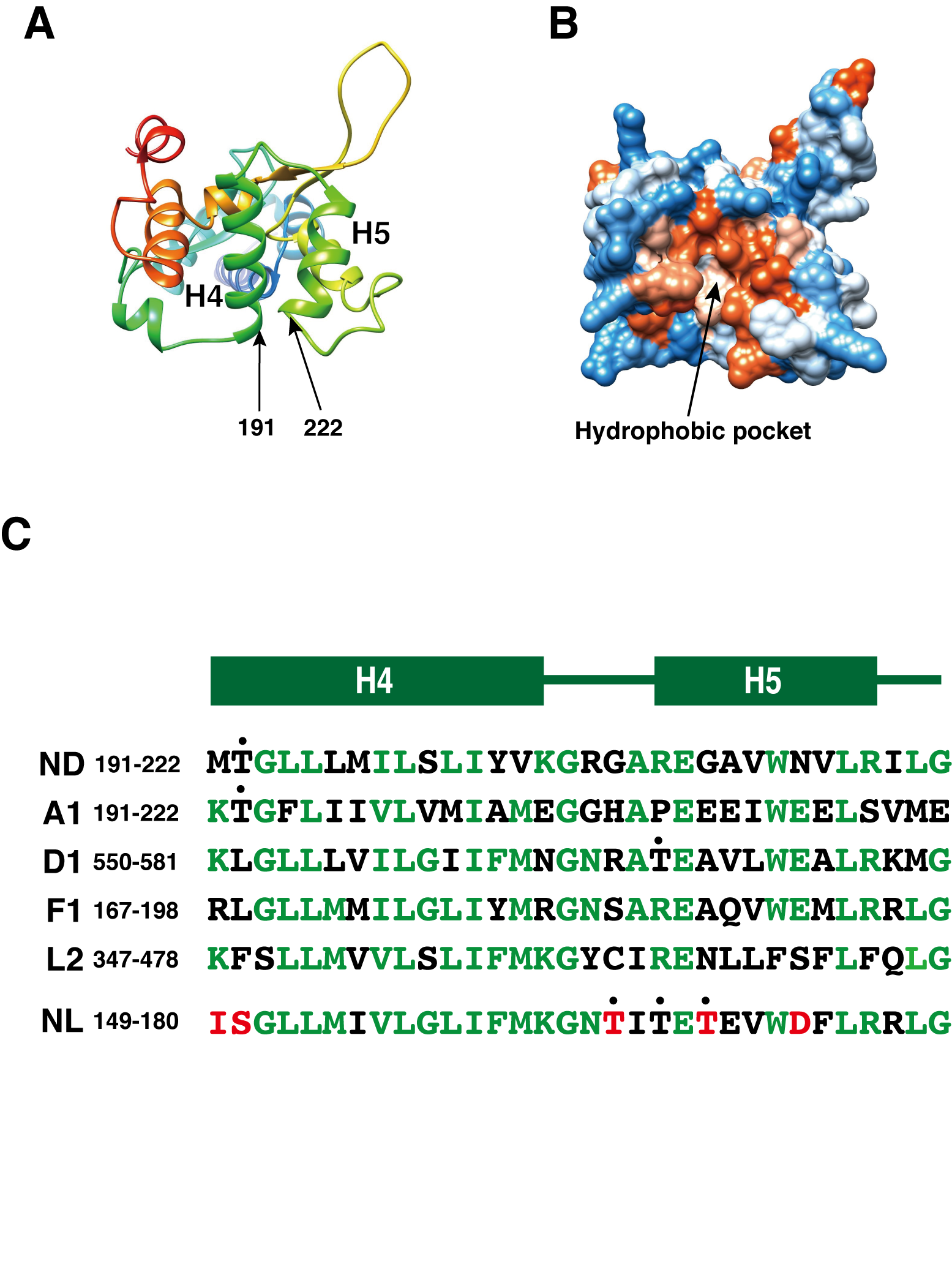

Supplement: Figure S4 — Protein structure modeling and primary sequence alignment of MAGE helices 4/5 regions. (A, B) Structure models of the necdin H4/5 region. A structural model of the necdin MHD was created by Spanner homology modeling program using the crystal structure data of necdin-like 2 (PDB 3NW0). A view focused on the H4/5 region (necdin aa 191-222) in the winged-helix B area is presented. Helices 4 (H4) and 5 (H5), positions of 191(M) and 222 (G) (A), the hydrophobic pocket consisting of surface hydrophobic (red) and hydrophilic (blue) residues (B) are indicated. (C) Sequence alignment of the H4/5 region. Consensus amino acid residues (≥3 identical)(green), the residues unique to necdin-like 2 (red), and Thr (T)(dot) are shown. Abbreviations: ND, necdin; A1, MAGEA1; D1, MAGED1; F1, MAGEF1; L2, MAGEL2; NL, necdin-like 2. (TIF) [file pone.0099503.s004.tif]
